# Did genome duplication drive the origin of teleosts? A comparative study of diversification in ray-finned fishes

**DOI:** 10.1186/1471-2148-9-194

**Published:** 2009-08-08

**Authors:** Francesco Santini, Luke J Harmon, Giorgio Carnevale, Michael E Alfaro

**Affiliations:** 1 Department of Ecology and Evolutionary Biology, University of California at Los Angeles, 651 Charles Young Dr. South, Los Angeles, CA 90095, USA; 2 Department of Biology, University of Idaho, Moscow, ID 83844, USA; 3 Dipartimento di Scienze della Terra, Universitá di Pisa, Via Santa Maria 53, 56126 Pisa, Italy; 4 Museo di Storia Naturale e del Territorio, Via Roma 79, 56011, Calci, Italy

## Abstract

**Background:**

One of the main explanations for the stunning diversity of teleost fishes (~29,000 species, nearly half of all vertebrates) is that a fish-specific whole-genome duplication event (FSGD) in the ancestor to teleosts triggered their subsequent radiation. However, one critical assumption of this hypothesis, that diversification rates in teleosts increased soon after the acquisition of a duplicated genome, has never been tested.

**Results:**

Here we show that one of three major diversification rate shifts within ray-finned fishes occurred at the base of the teleost radiation, as predicted by the FSGD hypothesis. We also find evidence for two rate increases that are much younger than the inferred age of the FSGD: one in the common ancestor of most ostariophysan fishes, and a second one in the common ancestor of percomorphs. The biodiversity contained within these two clades accounts for more than 88% of living fish species.

**Conclusion:**

Teleosts diversified explosively in their early history and this burst of diversification may have been caused by genome duplication. However, the FSGD itself may be responsible for a little over 10% of living teleost biodiversity. ~88% of species diversity is derived from two relatively recent radiations of freshwater and marine fishes where genome duplication is not suspected. Genome duplications are a common event on the tree of life and have been implicated in the diversification of major clades like flowering plants, vertebrates, and gnathostomes. However our results suggest that the causes of diversification in large clades are likely to be complex and not easily ascribed to a single event, even a dramatic one such as a whole genome duplication.

## Background

With approximately 28,872 species, teleost fishes constitute the dominant radiation of vertebrates on our planet [1]. One common explanation for this diversity is that a complete duplication of the entire genome [2] facilitated teleost diversification. This event is also known as the fish specific genome duplication, or FSGD [3]. Many studies have corroborated the occurrence of the genome duplication event [3-7], and several workers have hypothesized that the FSGD enabled the subsequent explosive diversification of teleosts by providing massive opportunities for evolutionary experimentation via gene duplication and decoupling [3-6]. This hypothesis, which we refer to as FSGD-FD (FSGD-facilitated diversification) has never been quantitatively tested.

The fact that teleosts contain over 99% of the total diversity found in ray-finned fishes might be taken as evidence that the diversification rate in teleosts is higher than in their close relatives. However a closer examination of species richness within teleosts reveals that the history of their diversification has been complex. Several teleost orders possess low species richness (Table 1), while the bulk of the biodiversity is concentrated in two large groups: the Ostariophysi, an almost exclusively freshwater clade that includes carps, danios, piranhas, and catfish, and the Perciformes (or perch-like fishes), a group of spiny-rayed fish that includes the majority of coastal and pelagic marine fish as well as some large freshwater lineages like cichlids and perches. Both of these groups appear in the fossil record 150–250 My after the estimated time of the FSGD [3,7,8]. One central prediction of the FSGD-FD hypothesis is that the diversification rate accelerated with the origin of teleosts. An alternative hypothesis is that major rate shifts are more recent, and correspond to the appearance of the species-rich teleost subclades described above. If true, this alternative hypothesis would suggest that the FSGD did not play a major role in generating fish biodiversity, due to the long interval between genome duplication and accelerated diversification.

**Table 1:** Ray-finned fish Species Richness

Lineage name in Fig. 4	Richness Fishbase [1]
Elasmobranchi	970

Latimeriidae	2

Dipnoi	3

Polypteriformes	18

Chondrostei	30

Holostei	8

Elopomorpha	924

Osteoglossomorpha	228

Clupeomorpha	382

Denticipidae	1

Gonorynchiformes	37

Cypriniformes	3665

Characiformes	1847

Siluriformes	3214

Gymnotiformes	148

Osmeriformes	44

Galaxiiformes	51

Stomiiformes	406

Argentiniformes	197

Salmoniformes	205

Esociformes	13

Myctophiformes	254

Aulopiformes	244

Percopsiformes+Gadiiformes	610

Polymixiiformes	10

Zeiformes	32

Lampriformes	24

Beryciformes [includes Stephanoberyciformes]	233

Ophidiiformes	460

Percomorpha	15639

Ray-finned fish species richness taken from Fishbase [1].

Recently, a number of molecular timescales for ray-finned fishes have been published. However most of these studies sampled a limited number of taxa and used relatively few fossil calibrations [7,9-11], making it difficult to date the origin of more than a few major actinopterygian crown groups. Here we present a large scale molecular timescale for actinopterygians that allows us to estimate the divergence times of most major lineages as well as the origin of many crown groups within them. We used this timescale along with information about taxonomic richness of unresolved actinopterygian clades to test the hypothesis that teleosts experienced an increase in diversification rates as predicted by the FSGD with a recently developed comparative method, MEDUSA (Modeling Evolutionary Diversification Using Stepwise AIC) ([16]; Additional file 1).

## Results

### Timetree

**Figure 1:**
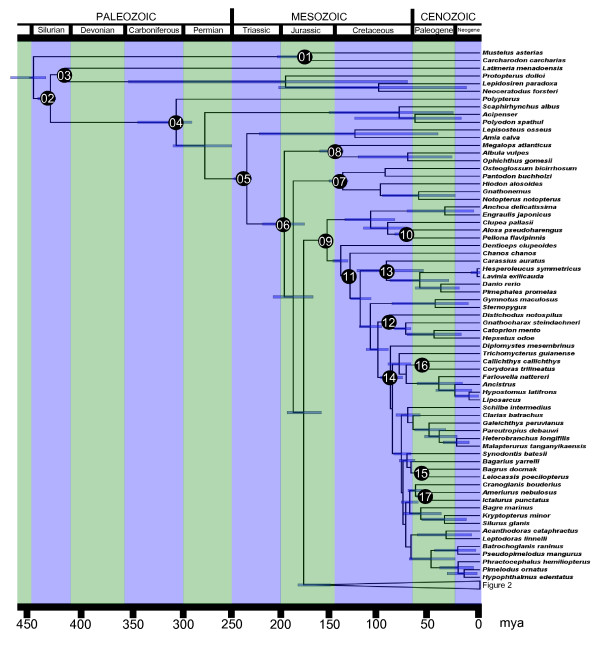
**Timetree of ray-finned fish**. Timetree of ray-finned fish based on 227 RAG1 sequences and 45 fossil calibration points. Includes taxa from Polypteriformes to Ostariophysi from Fig. 4.

**Figure 2:**
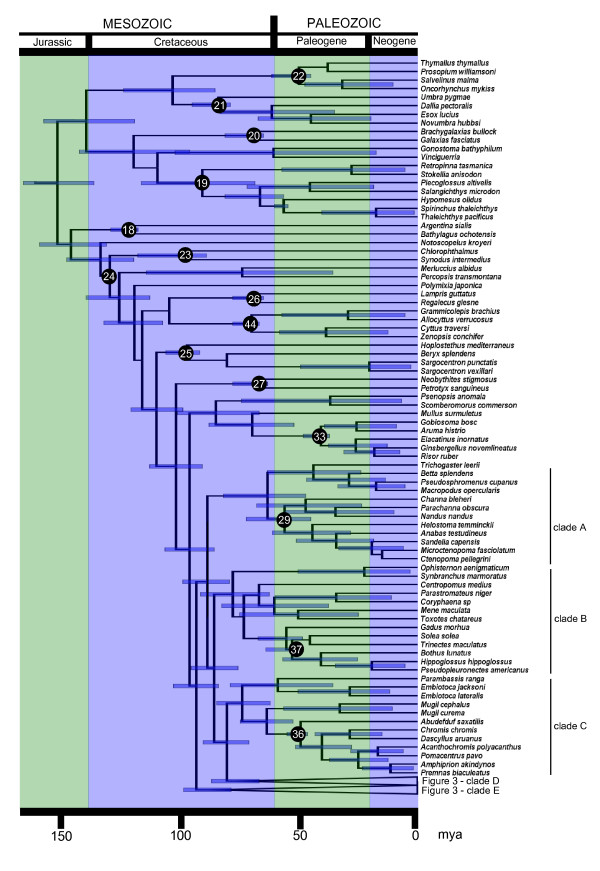
**Timetree of ray-finned fish**. Timetree of ray-finned fish based on 227 RAG1 sequences and 45 fossil calibration points. Includes taxa from Esociformes to part of Percomorpha from Fig. 4.

**Figure 3:**
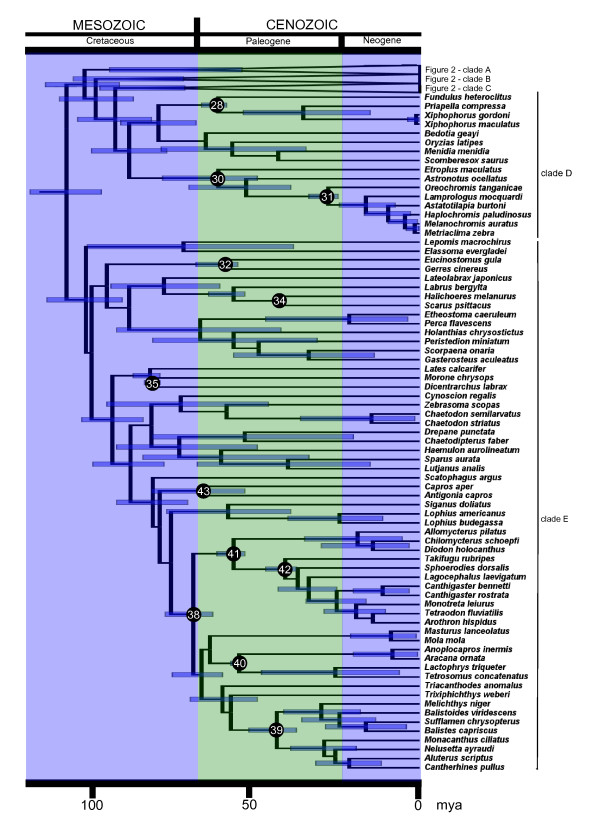
**Timetree of ray-finned fish**. Timetree of ray-finned fish based on 227 RAG1 sequences and 45 fossil calibration points. Includes part of Percomorpha from Fig. 4.

We downloaded and aligned 227 vertebrate RAG1 sequences from GenBank (221 actinopterygians, 4 sarcopterygians, 2 elasmobranchs, Additional File 2), and used Bayesian methods to infer divergence times with the ages of 44 clades constrained by fossils (Table 2). Our timetree (Fig 1, 2, 3) is the most comprehensive divergence time study of actinopterygians to date: it includes representatives of 39 of the 44 orders of ray-finned fish and 127 teleost families (which, taken together, represent over 80% of the total teleost species diversity); in addition, many of the 45 fossil calibration points used in this study, identified after a comprehensive review of the actinopterygian fossil record, have never before been integrated in a divergence time analysis (Additional file 3).

**Table 2:** Priors Used in Divergence Time Analysis

**Node**	**Name**	**Mean**	**95%**	**Prior**
	Root	428	505	

1	Elasmobranchii	16	41	Offset 165, means 1.9, SD 2.2

2	Osteichthyes	418	505	Offset 418, mean 1.21, SD 1.98

3	Sarcopterygii	407	505	offset 407, mean 1.49, SD 1.88

4	Actinopterygii	284	420	Lognormal, offset 284, mean 1.91, SD 1.83

5	Neopterygii	225	284	offset 225, mean 1.12, SD 1.8

6	Teleostei	152	228	offset 152, mean 1.30, SD 1.94

7	Osteoglossomorpha	130	152	offset 130, mean 1.0, SD 1.27

8	Elopomorpha	135	152	offset 135, mean 0.62, SD 1.35

9	Ostarioclupeomorpha	149	152	Offset 149, mean 0.1, SD 0.6

10	Pristigasteroidea	69	125	Offset 69, mean 1.48, SD 1.55

11	Ostariophysi	125	140	offset 125, mean 1.0, SD 1.04

12	Characiformes	68	100	Offset 68, mean 1.5, SD 1.2

13	Cyprinidae	49	100	Offset 49, mean 1.94, SD 1.2

14	Siluriformes	73	83.5	offset 73, mean 0.7, SD 1.0

15	Bagridae	59	73	Offset 59, mean 1.1, SD 0.94

16	Callichthyidae	55	73	Offset 55, mean 1.4, SD 0.94

17	Ictaluridae	56	73	Offset 56, mean 1.11, SD 1.05

18	Argentiniformes	127	152	Offset 127, mean 1.0, SD 1.35

19	Osmeridae	58.7	84	Offset 58.7, mean 1.08, SD 1.31

20	Galaxiidae	70	124	Offset 70, mean 1.01, SD 1.81

21	Esociformes	85	152	offset 85, mean 1.9, SD 1.4

22	Salmoniformes	48.6	125	offset 48.6, mean 1.54, SD 1.7

23	Aulopiformes	96	128	Offset 96, mean 1.5, SD 1.2

24	Acanthomorpha	99	122	Offset 99, mean 0.1, SD 1.85

25	Beryciformes	99	122	Offset 99, mean 1.5, SD 1.03

26	Lampriformes	70	98	Offset 70, mean 1.0, SD 1.42

27	Ophidiidae	68	98	Offset 68, mean 1.28, SD 1.29

28	Fundulidae vs Poeciilidae	55	99	Offset 55, mean 1.21, SD 1.55

29	Channoidea	48	84	Offset 48, mean 1.71, SD 1.14

30	Cichlidae	46	84	offset 46, mean 1.5, SD 1.3

31	African Cichlids	23.3	84	Offset 23.3, mean 1.26, SD 1.49

32	Gerreidae	52	84	offset 52, mean 1.16, SD 1.4

33	Gobiidae	40	84	offset 40, mean 1.37, SD 1.47

34	Labridae	50	84	offset 50, mean 0.9, SD 1.6

35	Moronidae	74	84	offset 74, mean 0.5, SD 1.1

36	Pomacentridae	50	84	offset 50, mean 1.24, SD 1.39

37	Pleuronectiformes	52	98	offset 52, mean 1.28, SD 1.55

38	Tetraodontiformes	59	98	offset 59, mean 0.8, SD 1.75

39	Balistoidea	35	50	offset 35, mean .9, SD 1.1

40	Ostracioidea	50	70	offset 50, mean 0.53, SD 1.5

41	Tetraodontoidea	50	70	Offset 50 mean 1.0, SD 1.22

42	Tetraodontidae	35	50	offset 35, mean 1.0, SD 1.04

43	Caproidae	50	99	offset 50, mean 1.51, SD 1.44

44	Zeiformes	72	98	offset 72, mean 1.01, SD 1.37

Among the Actinopterygii, the crown ray-finned fishes (Fig. 1, node 4) have a mean age of 298 Ma (with a 95% High Posterior Density, HPD: 284 to 337 Ma). This is approximately 100 My older than the oldest fossil, but also ~100 My younger than recent mitogenomic studies [9-11]. The key node in this study is the most recent common ancestor of teleosts (Fig. 1, node 6). We found that teleosts separated from their sister taxon, which in our analysis is a clade formed by gars and the bowfin (from here onwards we refer to this clade as Holostei) about 230 Ma, (95% HPD: 225–243 Ma), and radiated 193 Ma (95% HPD: 173–214 Ma). Our age estimate overlaps with the revised estimate for the FSGD of Hurley et al. [7] (226–316 Ma) but is not congruent with earlier estimates of 300–350 Ma based on less complete sampling [4,6,12]. The short fuse of 37 My between the origin of crown neopterygians and the origin of crown teleosts also suggests a relatively brief window of time for the occurrence of the FSGD. Within teleosts, we found that the two largest clades are both Cretaceous in origin. Crown Ostariophysi appeared 128 Ma (95% HPD: 125–134 Ma) (Fig. 1), and the crown Percomorpha (which differs from the Percomorpha of Nelson [13] because it includes also the Atherinomorpha, and includes over 50% of all teleosts) appeared 104 Ma (95% HPD: 93–115 Ma) (Fig. 2).

Our estimated ages for both the origin of the teleosts, as well as for the main splits among their major lineages (Table 3), are much younger than those inferred in mitogenomic studies [7,11], but are in fairly close agreement with dates provided by time-calibrated nuclear gene divergences in Hurley et al. [7]. This discrepancy might be due to an overall higher rate of evolution in mitochondrial genomes as discussed by Hurley et al. [7]. Within the more derived teleosts, our age estimates are generally younger than those previously published, but are in relatively good agreement with Inuoe et al. [9] for the origin of the acanthomorphs, with largely overlapping 95% HPDs in both studies. The age of two important percomorph clades, the cichlids and the tetraodontiforms, are drastically different between our study and previous work [10,11] (Fig. 3). The crown cichlids appear to have originated 57 Ma, with the split between the African and Neotropical lineages only 49 My old, dates that are consistent with those inferred by Genner et al. [14]. These ages appear to rule out a major role of the breakup of Gondwana [which dates to the Cretaceous] in determining the present distribution of this group. The Tetraodontiformes, the group that includes the pufferfish, an important model for vertebrate genomics, originated ~65 Ma, towards the end of the Cretaceous. This age is in agreement with the estimate based the previous analysis of a multigene dataset of Alfaro et al. [15], but is almost 100 Ma younger than Yamanoue et al.'s estimate based on mitogenomes [10].

**Table 3:** Divergence time estimates of focal ray-finned fish nodes

**Name of the split**	**Mean Age this study**	**95% age this study**	**Other study ages**
Condrichthyes vs. Osteichthyes	440		

MRCA of Neoselachii	178	165 to 200	

MRCA of Osteichthyes	423	418 to 435	415 to 524 [10]

MRCA of Sarcopterygii	409	407 to 415	

MRCA of Actinopterygii	299	284 to 337	397 to 478 mit [7]; 374 to 448 [9]

MRCA of Actinopteri	271	244 to 302	348 to 391 nuc [7]; 346 to 391 mit [7]; 337 to 413 [9]

MRCA of Neopterygii	230	225 to 243	295 to 372 nuc[7]; 327 to 378 mit [7]; 340 to 442 [10]

MRCA of Teleostei	193	173 to 214	268 to 326 mit [7]; 295 to 372 [9]

MRCA of Osteoglossomorpha	135	130 to 148	221 to 283 mit [7];

MRCA of Elopomorpha	140	135 to 158	210 to 272 mit [7];

MRCA of Ostarioclupeomorpha	151	149 to 153	192 to 255 mit [7]; 242 to 332 [10]; 204 to 275 [9]

MRCA of Clupeomorpha	108	84 to 133	

MRCA of Ostariophysi	128	125 to 134	

MRCA of Cypriniformes	92	56 to 123	

MRCA of Characiformes	80	68 to 84	

MRCA of Siluriformes	88	77 to 98	

MRCA of Euteleostei	164	147 to 180	182 to 244 mit [7]; 240 to 326 [10]; 197 to 267 [9]

MRCA of Salmoniformes	54	49 to 66	

MRCA of Esociformes	91	85 to 103	

MRCA of Galaxiiformes	74	70 to 87	

MRCA of Acanthomorpha	136	122 to 151	125 to 186 mit [7]; 191 to 264 [10]; 130 to 191 [9]

MRCA of Zeiformes	76	72 to 84	

MRCA of Lampridiformes	7	70 to 84	

MRCA of Beryciformes	105	99 to 114	

MRCA of Percomorpha	104	93 to 115	

MCRA of Caproidae	61	50 to 77	

MRCA of Cichlidae	57	46 to 73	72 to 108 [11]

African vs. American cichlids	49	37 to 66	

MRCA of Atherinomorpha	74	64 to 85	

MCRA of Pomacentridae	53	50 to 59	

MCRA of Moronidae	75	74 to 78	

MCRA of Labridae	53	50 to 60	

MCRA of Gobiidae	44	40 to 52	

MRCA of Tetraodontiformes	66	59 to 76	124 to 184 [10]

MRCA of Tetraodontidae	37	35.09, 41.06	55 to 86 [11]; 57 to 94 [10]

MRCA of Balistoidea	62	55.60, 71.78	95 to 146 [10]

MCRA of Pleuronectiformes	57	52 to 69	

Ages are in millions of years.

### Diversification rate study

**Figure 4:**
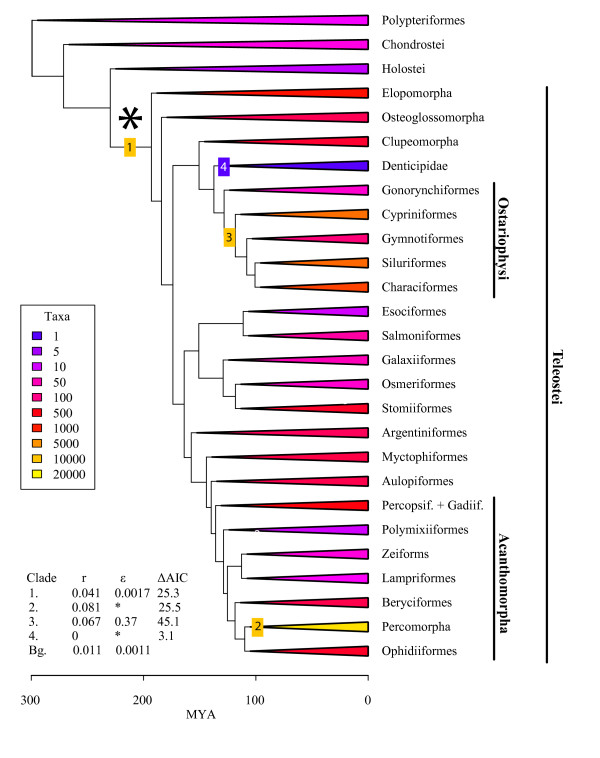
**Diversity tree for analyses of lineage diversification in ray-finned fish**. Diversity tree for analyses of lineage diversification in ray-finned fish. Clades from Fig. 1, 2, 3 are collapsed to 27 representative stem lineages and colored by extant species diversity. Clades with unusual diversification rates are denoted with numbers; yellow and blue numbers denote exceptionally fast and slow rates respectively, compared to background rates. Estimates for net diversification rate (r = b-d) and relative extinction rate (e = d/b) are included in the lower right table. Asterisk indicates FSGD event. Abbreviations is figure as follows: Percopsif.: Percopsiformes, Gadiif.: Gadiiformes.

To test whether diversification rate shifts supports the FSGD-diversification hypothesis, we applied a recently developed comparative method [MEDUSA, Modeling Evolutionary Diversification Using Stepwise AIC ([16], Additional file 1) to a 'diversity tree' derived from both the chronogram and species richness data compiled from the literature [Fig. 4]. Our stepwise procedure, based on the flexible rate model of Rabosky et al. [17], integrates both phylogenetic and taxonomic data [Fig. 4], and involves the assignment of rate shifts [both birth and death rates] to the optimal branches on the phylogeny with unresolved tips until additional rate changes do not substantially improve the AIC score. We tabulated the total species richness of actinopterygians, and partitioned it among representative stem lineages in our phylogeny. We pruned the chronogram down to representatives of each stem lineage with taxonomic information and estimated diversification statistics for the tree under the assumption of rate homogeneity across lineages. Then we tested for rate heterogeneity across lineages by implementing MEDUSA, to identify lineages representing significant departures from an expected background of diversification [16].

Our study reveals that actinopterygian biodiversity has been profoundly shaped by four diversification events. The most statistically significant of these occurred at the base of modern teleosts, as predicted by the FSGD-FD hypothesis, and involved a four-fold increase in net diversification rates [net rate r = birth rate – death rate] over the background rates estimated from the closest evolutionary relatives of teleosts. Additionally we find evidence for secondary rate increases in two lineages. The first of these preceded the radiation of percomorph fishes comprising most of the diversity of acanthomorphs or spiny-rayed fishes [13], including most of the coral reef-associated teleost families as well as most other marine fish diversity. The second increase preceded the radiation of a clade containing most ostariophysans, including the cypriniforms (carps and minnows), characiforms (pirhanas) and siluriforms (catfish). The final rate shift is a deceleration which gave rise to the denticled herring, the sole member of the family Denticipitidae, an ancient lineage that is the sister taxon of the Ostariophysi.

The teleost rate shift is characterized by a 3.7 fold increase in the rate of net diversification. Surprisingly, despite a net increase in diversification rate, estimated extinction rates in teleosts is higher than in nonteleosts (death rate, d_teleosts _= 6.98 × 10^-5^, d_nonteleosts _= 1.21 × 10^-5^). This contradicts suggestions that genome duplication in teleosts would have contributed to their diversification by making them more resistant to extinction [18]. Instead, turnover (e, the ratio of death to birth rate) is 1.5 times higher in teleosts than in non-teleosts. In comparison, the rate shift that gave rise to the percomorphs was less pronounced with a net diversification rate 1.98 times greater than the teleost rate. The rate shift leading to the ostariophysans reveals a period of increased volatility in the history of actinopterygians. Birth rates increased by more than 2.6 fold over teleost birth rates, but this rise in cladogenesis was checked by a substantial increase in extinction rates. This resulted in turnover rates in ostariophysans that were ~218 times higher than other teleosts. An increase in clade volatility may also have accompanied the increase in the percomorph diversification rate, but we were unable to calculate independent birth and death rates from the net diversification rate due to a lack of phylogenetic resolution within this large clade. In contrast to these three major rate accelerations, the shift underlying the denticled herring was characterized by a ~12 orders of magnitude decrease in net diversification rate. This result provides additional empirical evidence for the unusual nature of ancient clades of small size. These clades are too small and persist too long to be plausible outcomes under typical birth-death models unless the birth and death rates approach 0 [19,20].

## Discussion

Our study provides two lines of evidence in support of the FSGD-FD hypothesis. First, we find a significant increase in the diversification rate of teleosts. Second, the window in time between the split of teleosts and their sister taxon, the Holostei (230 Ma, 95% HPD:225–243 Ma, Fig. 1) and the subsequent radiation of crown teleosts (193 Ma, 95% HPD:173–214 Ma, Fig. 1) overlaps with the estimated age of the genome duplication itself (316-226 Ma) derived from dating of gene paralogs [7]. Furthermore, since teleost diversification is characterized by increases in the birth rate and not by decreases in the death rate (Fig. 4), our study suggests that genome-facilitated mechanisms of divergence, like lineage-specific nonfunctionalization [5,21], have played a larger role in teleost diversification than extinction resistance imparted by functional redundancy [18].

Our results also caution against the broad interpretation of the FSGD as the primary explanation for extant teleost diversity, of which approximately ~88% derives from the secondary diversification events in the percomophs and ostariophysans. One limitation of MEDUSA is that the assignment of rate shifts is limited to the level of phylogenetic resolution. Thus, the rate shifts leading to the ostariophysans and percomorphs might reflect a series of rate changes within these unresolved groups. As the teleost tree of life is uncovered, it will become possible to more precisely identify subclades or time intervals where diversification rates have changed. However given these caveats, we suggest that the radiation of teleosts is best understood as consisting of at least three pulses. Initial diversification may have been facilitated by mechanisms related to the FSGD [5,21], though further studies are needed to clarify how genome duplication can lead to sustained, elevated rates of diversification within a clade.

The second pulse (or series of pulses), the diversification within the largely freshwater ostariophysans, occurred about 100 My after the FSGD and coincides with the breakup of the supercontinent Gondwana during the Cretaceous. This geologic upheaval may have created opportunities for ecological diversification by creating new environments. Although many percomorph stem lineages also appear at this time, both the fossil record [8] and this molecular study suggest that the third pulse (or series of pulses) of teleost diversification occurred mostly in the Paleogene (65 to 23 Ma). Possible triggers of increased percomorph diversification include the establishment of scleractinian coral reefs and other tropical shallow water habitats like sea grasses [15,22], the fragmentation of the marine biotas due to geological events such as the progressive closing of the Tethys sea [23], sea-level fluctuations [24], and the establishments of steeper temperature gradients across the world's oceans, primarily due to the rearrangement of oceanic currents [25].

## Conclusion

Whole genome duplications are not uncommon in the tree of life and have been implicated in the diversification of other large clades, including most flowering plants (eudicots) and vertebrates [18,26]. Without quantitative comparison of diversification rates, however, it is difficult to identify the correlates of an evolutionary radiation, genomic or otherwise. The approach outlined here provides a mean for testing the central predictions of macroevolutionary hypotheses, including those linked to genome duplications, and represents an important step towards identifying the correlates of evolutionary radiations. Theoretical explanations of how genome duplication may lead to speciation have been proposed [27], and a number of examples are now known in which a genome duplication has been shown to have been linked to rounds of cladogenesis in yeasts and flowering plants [28,29]. However, additional work that elucidates the interplay between genomic isolating mechanisms and ecological opportunity is needed to more completely evaluate the role of genome duplication in shaping patterns of biodiversity.

## Methods

### Timetree inference

RAG1 sequences for 225 species of bony fish (including three species of lungfish, one species of coelacanths and 221 species of ray-finned fish), and two species of sharks, which we used as outgroups, were downloaded from GenBank (Additional file 2). The sampling was selected in order to both maximize the number of taxonomic groups that we could include in our analysis, and the number of fossil calibration points that could be assigned to the phylogeny. Sequences were aligned automatically using ClustallW [30], and the alignment was then refined by eye using MEGA 4 [31]. A survey of the fossil fish literature allowed us to identify 45 calibration points that were used to date 44 clades identified in the tree as well as the root of the tree (Additional file 3). We used BEAST v 1.4.6 [32] to estimate divergence times under a model of uncorrelated but log-normally distributed rates. We assigned soft upper bounds to the prior distributions of all fossil calibrations using log-normal distributions as described in Table 2. We specified a Yule prior on the rates of cladogenesis. The data set was assumed to have evolved under a GTR model with invariant sites and gamma-distributed rate heterogeneity. We constrained the monophyly of a number of groups in order to reflect generally accepted phylogenetic relationships. Five independent analyses of 20,000,000 generations each were run. Output from each run was analyzed using TRACER 1.4 [32]; 25% of the trees were discarded as burnin, and the remaining were combined using TreeAnnotator 1.4.6 to produce the timescale.

### Diversification rate analysis

MEDUSA [16] is an extension of the flexible rate shift model introduced by Rabosky et al. [17]. Rabosky's approach combines two likelihoods. The first is called the phylogenetic likelihood and uses the timing of splits along the resolved backbone of a phylogenetic tree to find maximum likelihood estimates for birth and death rates following equations developed by Nee et al. [33]. The second is called the taxonomic likelihood and uses information about the total species richness of an unresolved tip clade on a phylogeny along with the age of the split between the unresolved clade and its sister group to estimate diversification rates following methods developed by Magallon and Sanderson [34]. Rabosky et al. [17] presented a likelihood ratio test for a model where birth and death rates are allowed to shift on one branch of a phylogeny with unresolved tip clades to a model where birth and death rates are held constant across the tree. MEDUSA extends this procedure by adding rates in a stepwise fashion. First, the AIC score of a model with a single birth and death rate is calculated for the unresolved tree using the combined likelihood estimator presented by Rabosky et al. [17]. This two parameter model is then compared to the best four parameter model (two birth rates and two death rates) where the birth rate and the death rate are allowed to shift on the branch in the unresolved tree that produces the greatest improvement in the likelihood score. If the difference in AIC score between the two and four parameter models is substantial (ΔAIC ≥ 4, [35]) then this rate shift is retained. Next the four parameter model is compared to the best six parameter model by finding the optimal place on the tree for a third rate shift. The process is continued until additional rate shifts no longer produce a substantial improvement in AIC score. Full description of MEDUSA is present in Additional file 1.

To implement MEDUSA with the actinopterygian data, first we assembled taxonomic richness data from FISHBASE [1] for major lineages of fishes. Then we pruned the timetree in Fig. 1, 2, 3 down to 27 representative lineages. Our goal in pruning down the timetree was to preserve as much of the backbone of the timetree as would still permit us to assign species richness unambiguously to tip lineages. Thus, for example, we did not retain splitting events within Percomorpha because, although it was possible to assign species richness to some percomorph subclades such as tetraodontiforms, we could not confidently assign the entire species richness of other percomorphs to lineages included in our sampling. We used this pruned chronogram plus the taxonomic richness to estimate birth and death rates for ray-finned fishes and tested for rate shifts across the tree in R [36] using the LASER [37] and GEIGER [38] packages.

## Abbreviations

RAG1: recombination activating gene; FSGD: fish-specific whole-genome duplication event; MEDUSA: Modeling Evolutionary Diversification Using Stepwise AIC; Ma: million years ago; My: million years.

## Competing interests

The authors declare that they have no competing interests.

## Authors' contributions

FS and MEA contributed equally to this study and were responsible for study design, data analysis, and writing. FS downloaded and aligned sequences and assembled taxonomic richness data. FS and GC assigned fossil calibrations. MEA performed divergence time analysis. MEA and LJH performed diversification analysis. FS and GC wrote additional material. FS drew illustrations. All authors have read and approved the final manuscript.

## Supplementary Material

Additional file 1 **Description of MEDUSA.** Click here for file

Additional file 2 **GenBank accession numbers of sequences used in this study.** Click here for file

Additional file 3 **Description of timetree calibration points.** Click here for file
