# Supplementary material for: Did genome duplication drive the origin of teleosts? A comparative study of diversification in ray-finned fishes
Source: BMC Evol Biol. 2009 Aug 8;9:194. doi: 10.1186/1471-2148-9-194 (PMC2743667; doi:10.1186/1471-2148-9-194)

Did genome duplication drive the origin of teleosts? A comparative study of diversification in ray-finned fishes

Francesco Santini, Luke J. Harmon, Giorgio Carnevale, Michael E. Alfaro

Additional file 1 – Description of the MEDUSA method

MEDUSA (Modeling Stepwise Diversification Using Stepwise AIC) [1] is an extension of an approach described by Rabosky et al. [2] which uses phylogenetic and taxonomic information to estimate birth (b) and death (d) rates for an incompletely resolved phylogenetic tree. Nee et al. [3] describe a likelihood for estimating birth and death rates from the timing of splits in the resolved parts of a phylogeny. This phylogenetic likelihood from Nee et al. [3] is not useful in cases where the tree is not known. However, the timing of the split of an incompletely sampled clade from its sister group, along with an estimate of its species richness, does provide some information about diversification rates since all of the observed diversity in the clade must have arisen since the origin of the lineage [2, 4]. The net diversification rate (b - d) of a single unresolved stem lineage can be calculated using the stem group equations presented by Magallon and Sanderson [4]. The distribution of the age and species richness of multiple unresolved stem lineages such as those found in our diversity tree (Fig. 4), allows birth and death rates to be calculated using the taxonomic likelihood described by Rabosky et al. [2]. Using these equations, one can test whether a rate shift has occurred on any branch of the phylogeny by comparing likelihoods

or AIC scores from the simpler model with a single birth and death rate for the tree, to the more complex model where birth and death rates shift on one branch. This is described in Rabosky et al. [2].

MEDUSA extends this approach by using a stepwise procedure to add rate shifts to a tree until there is no substantial improvement in the AIC score. An example of this is shown in supplemental figure, Fig. S1. As a preliminary step we compare the fit of a birth-death model to pure birth model using AIC scores. In our example, this reveals overwhelming support for the birth-death model (ΔAIC > 146) (Fig. S1B). Then we calculate the AIC score for a four parameter model where b and d are allowed to shift on the optimal location of the phylogeny. To find the optimal branch for a rate shift, we allow b and d to change along every branch of the tree in Fig. S1A and retain the model that yields the greatest improvement in AIC score. In this case, a rate shift along the branch leading to the species-rich clade 'E' is selected. We compare the AIC score of this four-rate model to the original two-rate birth death model and accept the four rate model if the improvement in AIC score is substantial (ΔAIC > 4 [5]). In Fig S1, a rate shift on the branch leading to clade 'E' yields an improvement in AIC score of ~6, so the four-rate model is retained. We repeat this process and compare the four-parameter model (with a rate shift leading to clade 'E') to the optimal six parameter model (a background b-d rate, a shift in b and d on the branch leading to E, and a second rate shift on the optimal branch in the phylogeny. In our example, allowing the rate to shift again on clade 'C' nets the largest

improvement in AIC. This improvement is substantial (ΔAIC > 19) so the three rate shift model is retained. Additional rate shifts result in minor improvements to the AIC score or cause it to get worse, so we stop after allowing rates to shift on clade 'C'

References

1. Alfaro ME, Santini F, Brock CD, Alamillo H, Dornburg A, Rabosky DL, Carnevale G, Harmon LJ: **Lineage-specific Diversification Rates and High Turnover in the History of Jawed Vertebrates.** *PNAS, in press.*

2. Rabosky DL, Donnellan SC, Talaba AL, Lovette IJ: **Exceptional among-lineage variation in diversification rates during the radiation of Australia's most diverse vertebrate clade.** *Proc R Soc London, Ser B* 2007, **274**:2915-2923.

3. Nee S, May RM, Harvey PH: **The reconstructed evolutionary process.** *Phil Trans Roy Soc (Lond) B* 1994, **344**:305-311.

4. Magallon S, Sanderson MJ: **Absolute diversification rates in angiosperm clades.** *Evolution* 2001, **55**:1762-1780.

5. Burnham KP, Anderson DR: **Model selection and multimodel inference, a practical information-theoretic approach.** York: Springer; 2003.

Supplemental figure S1- MEDUSA

S1. MEDUSA example. A) Diversity tree with location of rate shifts identified by MEDUSA (black bars). B) Improvement in AIC score of rate-shift models.

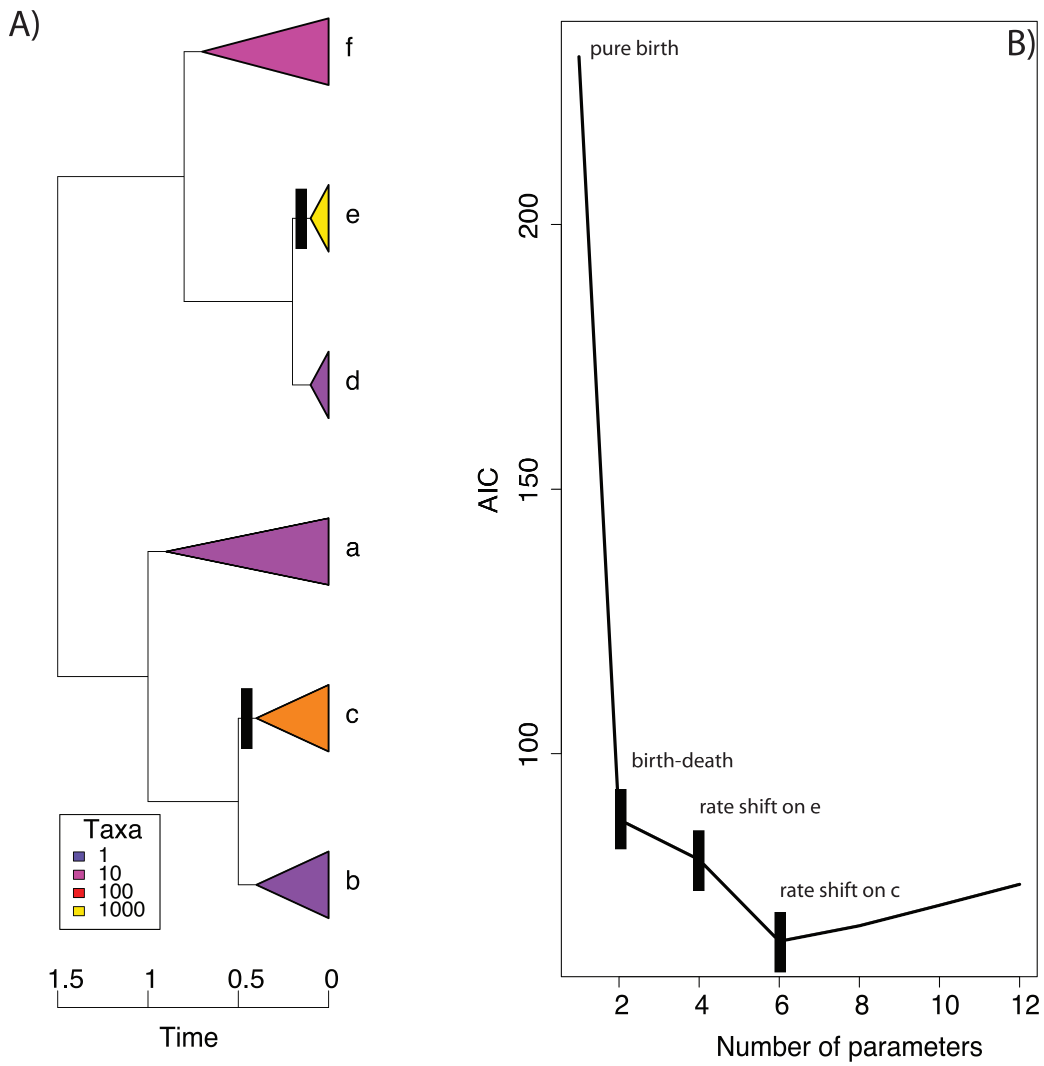

Supplement: Additional file 1 — Description of MEDUSA. [file 1471-2148-9-194-S1.pdf]
