# Supplementary material for: Did genome duplication drive the origin of teleosts? A comparative study of diversification in ray-finned fishes
Source: BMC Evol Biol. 2009 Aug 8;9:194. doi: 10.1186/1471-2148-9-194 (PMC2743667; doi:10.1186/1471-2148-9-194)
Supplement: Additional file 2 — GenBank accession numbers of sequences used in this study. [file 1471-2148-9-194-S2.pdf]

Did genome duplication drive the origin of teleosts? A comparative study of diversification in ray-finned fishes

Francesco Santini, Luke J. Harmon, Giorgio Carnevale, Michael E. Alfaro

Additional file 2 – GenBank accession numbers of sequences used in this study

Table S1 - List of taxa and Rag1 GenBank accession numbers included in study.

GenBank sequence #	Taxon	Common name	Family	Order	Lineage taxon belongs to in Fig. 4
AF135482	*Carcharodon carcharias*	Great white shark	Lamnidae	Lamniformes	
AY462188	*Mustelus asterias*	Starry smooth-hound shark	Triakidae	Carchariniformes	
AY442926	*Lepidosiren paradoxa*	South American lungfish	Lepidosirenidae	Lepidosireniformes	
AY442928	*Protopterus dolloi*	African lungfish	Protopteridae		
AY442927	*Neoceratodus forsteri*	Australian lungfish	Ceratodontidae	Ceratodontiformes	
	Latimeria chalumnae	Coelacanth	Latimeriidae	Coelacanthiformes	
AY442925	*Latimeria menadoensis*	Coelacanth			
AF369055	*Polypterus sp.*	Bichir	Polypteridae	Polypteriformes	Polypteriformes
AF369058	*Lepisosteus osseus*	Longnose gar	Lepisosteidae	Lepisosteiformes	Holostei
AY430199	*Amia calva*	Bowfin	Amiidae	Amiiformes	Holostei
AF369056	*Acipenser sp.*	sturgeon	Acipenseridae	Acipenseriformes	Chondrostei
AY430198	*Scaphirhynchus albus*	Pallid sturgeon			
U15613 AF369057	*Polyodon spathula*	Mississippi paddlefish	Polyodontidae		
AY430200	*Hiodon alosoides*	Goldeye	Hiodontidae	Osteoglossiformes	Osteoglossomorpha
AF369062	*Gnathonemus sp.*	Elephantfishes	Mormyridae		
AF369063	*Notopterus notopterus*	Bronze featherback	Notopteridae		
AY430201	*Osteoglossum bicirrhosum*	Arawana	Osteoglossidae		
AF369061	*Pantodon buchholzi*	Freshwater butterflyfish	Pantodontidae		
AY430202	*Albula vulpes*	Bonefish	Albulidae	Albuliformes	Elopomorpha

AY430204	*Megalops atlanticus*	Tarpon	Megalopidae	Elopiformes	
AY430203	*Ophichthus gomesii*	Shrimp eel	Ophichthidae		
DQ912115	*Alosa pseudoharengus*	Alewife	Clupeidae	Clupeiformes	Clupeomorpha
DQ912118	*Clupea pallasii*	Pacific herring			
DQ912100	*Denticeps clupeoides*	Denticle herring	Denticipitidae		Denticipitidae
DQ912108	*Anchoa delicatissima*	Slough anchovy	Engraulidae		Clupeomorpha
AY430205	*Engraulis japonicus*	Japanese anchovy			
AY430206	*Pellona flavipinnis*	Yellowfin river pellona	Pristigasteridae		
AY430212	*Catoprion mento*	Wimple piranha	Characidae	Characiformes	Characiformes
AY430211	*Gnathocharax steindachneri*				
DQ492425	*Distichodus notospilus*		Citharinidae		
DQ912097	*Hepsetus odoe*	Kafue pike	Hepsetidae		
U15614	*Carassius auratus*	Goldfish	Cyprinidae	Cypriniformes	Cypriniformes
U71093	*Danio rerio*	Zebrafish			
AY059468	*Hesperoleucus symmetricus*	California roach			
AY059469	*Lavinia exilicauda*	Hitch			
AY430210	*Pimephales promelas*	Fathead minnow			
AY430207	*Chanos chanos*	Milkfish	Chanidae	Gonorynchiformes	Gonorynchiformes
AY359225	*Gymnotus sp.*	Knifefish	Gymnotidae	Gymnotiformes	Gymnotiformes
DQ492426	*Sternopygus sp.*	Glass knifefishes	Sternopygidae		
DQ492524	*Bagre marinus*	Gafftopsail sea catfish	Ariidae	Siluriformes	Siluriformes
DQ492527	*Galeichthys peruvianus*	Peruvian sea catfish			
DQ492458	*Bagrus docmak*	Semutundu	Bagridae		
DQ492457	*Leiocassis poecilopterus*				
DQ492436	*Callichthys callichthys*	Cascarudo	Callichthyidae		
DQ492437	*Corydoras trilineatus*	Threestripe corydoras			
DQ492521	*Clarias batrachus*	Walking catfish	Clariidae		
DQ492520	*Heterobranchus longifi*	Vundu			
DQ492514	*Cranoglanis bouderius*		Cranoglanididae		
DQ492428	*Diplomystes mesembrinus*		Diplomystidae		
DQ492466	*Acanthodoras cataphractus*	Spiny catfish	Doradidae		
DQ492468	*Leptodoras linnelli*				
AY430209	*Ameiurus nebulosus*	Brown bullhead	Ictaluridae		
DQ492511	*Ictalurus punctatus*	Channel catfish			
AY552046	*Ancistrus sp.*	Armored catfishes	Loricaridae		
DQ492441	*Farlowella nattereri*	Armored catfishes			
AY552038	*Hypostomus latifrons*	Armored catfishes			

DQ492443	*Liposarcus multiradiatus*	Armored catfishes			
DQ492498	*Malapterurus tanganyikaensis*		Malapteruridae		
DQ492495	*Synodontis batesii*	Upsidedown catfish	Mochokidae		
DQ492474	*Hypophthalmus edentatus*	Highwaterman catfish	Pimelodidae		
DQ492476	*Phractocephalus hemioliopterus*	Redtail catfish			
DQ492475	*Pimelodus ornatus*				
DQ492473	*Batrochoglanis raninus*		Pseudopimelodidae		
DQ492472	*Pseudopimelodus mangurus*				
DQ492507	*Pareutropius debauwi*	African glass catfish	Schilbeidae		
DQ492508	*Schilbe intermedius*	Silver catfish			
DQ492486	*Kryptopterus minor*		Siluridae		
AY552051	*Silurus glanis*	Wels catfish			
DQ492446	*Bagarius yarrelli*	Goonch	Sisoridae		
DQ492431	*Trichomycterus guianense*	Pencil or parasitic catfishes	Trichomycteridae		
AY380542	*Esox lucius*	Northern pike	Esocidae	Esociformes	Esociformes
AY380540	*Dallia pectoralis*	Alaska blackfish	Umbridae		
AY380546	*Novumbra hubbsi*	Olympic mudminnow			
AY380549	*Umbra pygmae*	Eastern mudminnow			
AY430228	*Argentina sialis*	North-Pacific argentine	Argentinidae	Argentiniformes	Argentiniformes
AY443564	*Bathylagus ochotensis*	Eared blacksmelt	Bathylagidae		
AY430219	*Brachygalaxias bullocki*	Galaxias	Galaxiidae	Galaxiiformes	Galaxiiformes
AY430218	*Galaxias fasciatus*	Banded kokopu			
AY380538	*Hypomesus olidus*	Pond smelt	Osmeridae	Osmeriformes	Osmeriformes
AY430215	*Spirinchus thaleichthy*	Longfin smelt			
AY380537	*Thaleichthys pacificus*	Eulachon			
AY380536	*Plecoglossus altivelis*	Ayu	Plecoglossidae		
AY430216	*Retropinna tasmanica*	Tasmanian smelt	Retropinnidae		
AY430217	*Stokellia anisodon*	Stokell's smelt			
AY380539	*Salangichthys microdon*	Japanese icefish	Salangidae		
U15663	*Oncorhynchus mykiss*	Rainbow trout	Salmonidae	Salmoniformes	Salmoniformes
AY430213	*Prosopium williamsoni*	Mountain whitefish			
AY380535	*Salvelinus malma*	Dolly varden			
AY430214	*Thymallus_thymallus*	Grayling			
AY438703	*Gonostoma bathyphilum*	Bristlemouths	Gonostomatidae	Stomiiformes	Stomiiformes
AY442363	*Vinciguerria sp.*	Lightfishes	Phosichthyidae		
AY430220*	*Chlorophthalmus agassizi*	Greeneyes	Chlorophthalmidae	Aulopiformes	Aulopiformes

AY308763	*Synodus intermedius*	Sand diver	Synodontidae		
AY430221	*Notoscopelus kroyeri*	Lancet fish	Myctophidae	Myctophiformes	Myctophiformes
AY308764	*Lampris guttatus*	Opah	Lampridae	Lampriformes	Lampriformes
AY430222	*Regalecus glesne*	King of herrings	Regalecidae		
AY308765	*Polymixia japonica*	Silver eye	Polymixiidae	Polymixiiformes	Polymixiiformes
EF033043	*Neobythites stigmosus*		Ophidiidae	Ophidiiformes	Ophidiiformes
AY308782	*Petrotyx sanguineus*	Redfin brotula			
AY308766	*Percopsis transmontana*	Sand roller	Percopsidae	Percopsiformes	Percopsiformes + Gadiiformes
EF095636	*Beryx splendens*	Splendid alfonsino	Berycidae	Beryciformes	Beryciformes
AY430223	*Sargocentron punctatissimum*	Speckled squirrelfish	Holocentridae		
AY308770	*Sargocentron vexillarium*	Dusky squirrelfish			
EF095635	*Hoplostethus mediterraneus*	Mediterranean slimehead	Trachichthyidae		
AF369064	*Gadus morhua*	Atlantic cod	Gadidae	Gadiformes	Percopsiformes + Gadiiformes
AY308787	*Merluccius albidus*	Offshore hake	Merlucciidae		
AY308786	*Lophius americanus*	American angler	Lophiidae	Lophiiformes	Percomorpha
EF095637	*Lophius budegassa*	Black-bellied angler			
EF095640	*Bedotia geayi*	Red-Tailed Silverside	Bedotiidae	Atheriniformes	
AY430225	*Menidia menidia*	Atlantic silverside	Atherinopsidae		
AB120889	*Oryzias latipes*	Japanese rice fish	Adrianichthyidae	Beloniformes	
EF095641					
AY308771	*Scomberesox saurus*	Atlantic saury	Scomberesocidae		
EF033040	*Fundulus heteroclitus*	Mummichog	Fundulidae	Cyprinodontiformes	
DQ235860	*Priapella compressa*	Palenque priapella	Poeciliidae		
DQ235866	*Xiphophorus gordoni*	Northern platyfish			
DQ235880	*Xiphophorus maculatus*	Southern platyfish			
AF369065 EF095639	*Mugil cephalus*	Flathead mullet	Mugilidae	Mugiliformes	
AY308783	*Mugil curema*	White mullet			
EF033039	*Gasterosteus aculeatus*	Threespine stickleback	Gasterosteidae	Gasterosteiformes	
AY308776	*Zebrasoma scopas*	Twotone tang	Acanthuridae	Perciformes	
EF095646	*Parambassis ranga*	Indian glassy fish	Ambassidae		
AY763773	*Anabas testudineus*	Climbing perch	Anabantidae		
AY763776	*Ctenopoma pellegrini*	ctenopoma			
AY763777	*Microctenopoma fasciolatum*	Banded ctenopoma			
AY763778	*Sandelia capensis*	Cape kurper			
AY308785	*Antigonia capros*	Deepbody boarfish	Caproidae		
EF095638	*Capros aper*	Boarfish			

EF095654	*Parastromateus niger*	Black pomfret	Carangidae
AY430227	*Lepomis macrochirus*	Bluegill sunfish	Centrarchidae
EF095677	*Psenopsis anomala*	Melon seed	Centrolophidae
EF095649	*Centropomus medius*	Blackfin snook	Centropomidae
EF095655	*Chaetodon semilarvatus*	Bluecheek butterflyfish	Chaetodontidae
AY308775	*Chaetodon striatus*	Banded butterflyfish	
AY763786	*Channa bleheri*	Snake-head	Channidae
AY763788	*Parachanna obscura*	Snake-head	
DQ012245	*Astatotilapia burtoni*	Cichlid	Cichlidae
EF095671	*Astronotus ocellatus*	Oscar	
EF095672	*Etroplus maculatus*	Orange chromide	
DQ012233	*Haplochromis paludinos*	Cichlid	
DQ012226	*Lamprologus mocquardi*	Cichlid	
DQ012220	*Melanochromis auratus*	Golden mbuna	
DQ012249	*Metriaclima zebra*	Zebra mbuna	
DQ012223	*Oreochromis tanganicae*	Cichlid	
EF095653	*Coryphaena sp.*	dolphinfish	Coryphaenidae
AY308772	*Drepane punctata*	Spotted sicklefish	Drepaneidae
AY308784	*Elassoma evergladei*	Everglades pygmy sunfish	Elassomatidae
EF095670	*Embiotoca jacksoni*	Black perch	Embiotocidae
AY208615	*Embiotoca lateralis*	Striped seaperch	
AY308773	*Chaetodipterus faber*	Atlantic spadefish	Ephippidae
EF095663	*Eucinostomus gula*	Jenny mojarra	Gerreidae
EF095666	*Gerres cinereus*	Yellow fin mojarra	
AY846564	*Aruma histrio*	Slow goby	Gobiidae
AY846500	*Elacatinus inornatus*	Inornate goby	
AY846511	*Ginsburgellus novemlineatus*	Nineline goby	
AY846562	*Gobiosoma bosc*	Naked goby	
AY846561	*Risor ruber*	Tusked goby	
EF095661	*Haemulon aurolineatum*	Tomtate grunt	Haemulidae
AY763779	*Helostoma temminckii*	Kissing gourami	Helostomatidae
AY208617	*Halichoeres melanurus*	Tail-spot wrasse	Labridae
EF095669	*Labrus bergylta*	Ballan wrasse	
AF369066	*Lates calcarifer*	Barramundi	Latidae
EF033042	*Lutjanus analis*	Mutton snapper	Lutjanidae
EF095659	*Mene maculata*	Moonfish	Menidae
EF095651	*Dicentrarchus labrax*	European seabass	Moronidae
EF095650	*Lateolabrax japonicus*	Japanese seaperch	

AY308767	*Morone chrysops*	White bass		
EF095658	*Mullus surmuletus*	Striped red mullet	Mullidae	
AY330979	*Nandus nandus*	Gangetic leaffish	Nandidae	
AF519728	*Betta splendens*	Siamese fighting fish	Osphronemidae	
AF519737	*Macropodus opercularis*	Paradise fish		
AF519738	*Pseudosphromenus_cupanus*	Spiketail paradisefish		
AF519734	*Trichogaster leerii*	Pearl gourami		
AY430226	*Etheostoma caeruleum*	Rainbow darter	Percidae	
AY308768	*Perca flavescens*	Yellow perch		
AY208624	*Abudefduf saxatilis*	Sergeant major	Pomacentridae	
AY208625	*Acanthochromis polyacanthus*	Spiny chromis		
AY208628	*Amphiprion akindynos*	Barrier reef anemonefish		
AY208640	*Chromis chromis*	Damselfish		
AY208649	*Dascyllus aruanus*	Whitetail dascyllus		
EF095673	*Pomacentrus pavo*			
AY208632	*Premnas biaculeatus*	Spinecheek anemonefish		
EF095675	*Scarus psittacus*	Common parrotfish	Scaridae	
EF095668	*Scatophagus argus*	Spotted scat	Scatophagidae	
EF095660	*Cynoscion regalis*	Gray weakfish	Sciaenidae	
EF095676	*Scomberomorus commerson*	Narrow-barred Spanish mackerel	Scombridae	
EF095645	*Holanthias chrysostictus*		Serranidae	
AY308777	*Siganus doliatus*	Barred spinefoot	Siganidae	
EF095657	*Sparus aurata*	Gilthead seabream	Sparidae	
EF095652	*Toxotes chatareus*	Spotted archerfish	Toxotidae	
AY430224	*Trinectes maculatus*	Hogchoker	Achiridae	Pleuronectiformes
AY308769	*Bothus_lunatus*	Plate fish	Bothidae	
AY454396	*Hippoglossus hippoglossus*	Atlantic halibut	Pleuronectidae	
AF369067	*Pseudopleuronectes americanus*	Winter flounder		
EF095644	*Solea solea*	Common sole	Soleidae	
AY308774	*Peristedion miniatum*	Armored searobin	Peristediidae	Scorpaeniformes
EF095642	*Scorpaena onaria*		Scorpaenidae	
AY359221	*Ophisternon aenigmaticum*	Obscure swamp eel	Synbranchidae	Synbranchiformes
AY359218	*Synbranchus marmoratus*	Marbled swamp eel		
AY700346	*Anoplocapros inermis*	Eastern smooth boxfish	Aracanidae	Tetraodontiformes
AY700348	*Aracana ornata*	Ornate cowfish		

AY700308	*Balistes capriscus*	Grey triggerfish	Balistidae		
AY700320	*Balistoides viridescens*	Titan triggerfish			
AY700313	*Melichthys niger*	Black triggerfish			
AY700321	*Sufflamen chrysopterus*	Halfmoon triggerfish			
AY700324	*Allomycterus pilatus*	Deepwater burrfish	Diodontidae		
AY700325	*Diodon holocanthus*	Long-spine porcupinefish			
AY700326	*Chilomycterus schoepfi*	Striped burrfish			
AY308792	*Masturus lanceolatus*	Sharptail mola	Molidae		
AY700328	*Mola mola*	Ocean sunfish			
AY700331	*Aluterus scriptus*	Scrawled filefish	Monacanthidae		
AY700333	*Cantherhines pullus*	Orangespotted filefish			
AY700334	*Monacanthus ciliatus*	Fringed filefish			
AY700340	*Nelusetta ayraudi*	Chinaman-leatherjacket			
AY700344	*Lactophrys triqueter*	Smooth trunkfish	Ostraciidae		
AY308794	*Tetrosomus concatenatus*	Triangular boxfish			
AY700367	*Arothron hispidus*	White-spotted puffer	Tetraodontidae		
AY700358	*Canthigaster bennetti*	Bennett's sharpnose puffer			
AY700351	*Canthigaster rostrata*	Caribbean sharpnose-puffer			
AY700365	*Lagocephalus laevigatus*	Smooth puffer			
AY700364	*Monotreta leiurus*				
AY308795	*Sphoeroides dorsalis*	Marbled puffer			
AF108420	*Takifugu rubripes*	Torafugu			
AY700355	*Tetraodon fluviatilis*	Green pufferfish			
AY308789	*Trixiphichthys weberi*	Blacktip tripodfish	Triacanthidae		
AY308788	*Triacanthodes anomalus*	Spikefish	Triacanthodidae		
AY308779	*Cyttus traversi*	King dory	Cyttidae	Zeiformes	Zeiformes
AY308780	*Grammicolepis brachiusculus*	Thorny tinselfish	Grammicolepididae		
AY308781	*Allocyttus verrucosus*	Warty oreo	Oreosomatidae		
AY308778	*Zenopsis conchifer*	Silvery John dory	Zeidae		
