# Supplementary material for: Did genome duplication drive the origin of teleosts? A comparative study of diversification in ray-finned fishes
Source: BMC Evol Biol. 2009 Aug 8;9:194. doi: 10.1186/1471-2148-9-194 (PMC2743667; doi:10.1186/1471-2148-9-194)
Supplement: Additional file 3 — Description of timetree calibration points. [file 1471-2148-9-194-S3.pdf]

Did genome duplication drive the origin of teleosts? A comparative study of diversification in ray-finned fishes

Francesco Santini, Luke J. Harmon, Giorgio Carnevale, Michael E. Alfaro

Additional files

Additional file 3 – Description of timetree calibration points

To obtain a molecular timetree, we assigned fossil calibrations to the root and 44 additional nodes on the Bayesian consensus tree (Fig. 1-3). For each constraint below, the age of the fossil provided a hard lower bound on the minimum age of the node. Upper limits on the ages of constrained nodes are generally difficult to determine. We used log normal priors in our BEAST analysis because this distribution allowed us to express our prior belief, based on the occurrence of related taxa in the fossil record, of the maximum age of each constraint as a 95% cumulative probability while still allowing for the possibility that a constrained node could be older. Thus, fossils in our analysis provided hard lower and soft upper bounds. Justification for these bounds for each constraint is given below.

Age of the Root between Osteichthyes and Outgroups. The sister group of the Osteichthyes (bony fishes) is currently unknown. It may be represented by the Chondrichthyes (cartilaginous fishes), or by the Acanthodii. The fossil record of the Chondrichthyes dates to at least the early-middle Caradoc of the Harding Sandstone,

Colorado (Ordovician, 456-454 Ma) [1]. The acanthodian *Onchus clintonii* dates to the Sheinwoodian and Homerian (Silurian, 436-428 Ma)2 [2]. Due to the uncertainty in the sister group relationships between Osteichthyes, Acanthodii, and Chondrichthyes, we assigned to the root a minimum age of 428 My. Placing an upper limit on the root is problematic, due the uncertain relationships of many worm-like Cambrian fossils that have been tentatively referred to as chordates. *Pikaia*, from the Burgess Shale fossil deposits of the late Cambrian of British Columbia, Canada (505 Ma) is a prevertebrate that has been identified as probably being related to cephalochordates [3]. Our prior uses the age of these deposits to set the 95% confidence interval.

Elasmobranchii (Lamnidae vs. Scyliorhinidae) (Fig. 1, node 1). While Elasmobranchii (sharks) have a fossil record that spans back to the Ordovician, crown sharks are much more recent, having appeared in the fossil record during the Mesozoic. The earliest fossils that can be used to date the split between the two species that we used in this analysis is an unnamed and undescribed taxon from deposits of the Bathonian (Jurassic, 167.7-164.7 Ma), which was assigned to the family Scyliorhinidae [4]. Our prior assumed a minimum age of 165 My, and placed 95% of the weight on divergence times within the time span marked by the first Elasmobranchii (415 Ma) [4].

MRCA of Osteichthyes (Sarcopterygii vs. Actinopterygii) (Fig. 1, node 2). The oldest known fossil from this clade is the stem actinopteygian *Andreolepis hedei* from the Lludlowian of Gotland, Sweden (Silurian, 422-418 Ma) [5]. Our prior assumed a

minimum age of 418 My, and used the age of the Burgess Shale deposits (505 My) for the 95% upper limit.

MRCA of Sarcopterygii (Dipnotetrapodomorpha vs. Coelacanthimorpha) (Fig. 1, node 3). The oldest sarcopterygian is *Psarolepis romeri* from the Lludlow-Pridoli of China (Silurian, 420-418 Ma), but this is likely a stem taxon. The oldest taxon that marks the split between coelacanths, lungfishes, and tetrapods is *Eoactinistia foreyi*, the oldest coelacanthimorph from the Pragian (Devonian, 409-407 Ma) [6]. The prior thus assumed 407 My as the minimum age of the split, and a 95% upper bound of 505 My (reflecting the upper age of the Burgess shale deposit).

MRCA of Actinopterygii (Actinistia vs Acipenseriformes vs Neoteleostei) (Fig. 1, node 4). The oldest fossil known to belong to this clade is the stem actinopteygian *Andreolepis hedei* from the Lludlowian of Gotland, Sweden (Silurian, 422-418 Ma) [5]. The oldest taxon that can be assigned to the crown is the neopterygian *Brachydegma caelatum*, from the Artkinsian of Texas (early Permian, 284 Ma) [7]. Our prior thus assumed 284 My as the minimum age, and 418 My for the upper bound.

MRCA of Neopterygii (Amiiformes vs Lepisosteiformes vs Teleostei) (Fig. 1, node 5). The oldest crown taxon is the stem teleost *Pholidophoretes salvus*. The oldest stem is *Brachydegma caelatum*, from the Artkinsian of Texas (early Permian, 284 Ma) [7]. Our prior thus assumed 225 My as the minimum age, and 284 My for the upper bound.

MRCA of Teleostei (Elopomorpha vs Osteoglossomorpha vs Clupeocephala) (Fig. 1, node 6). The oldest taxon assigned to this clade is *Anaethalion*, from the late Kimmeridgian lithographic limestone of Nusblingen, Germany, and Cerin, France (Jurassic, 152 My) [8]. The oldest stem teleost is *Pholidophoretes salvus* (Pholidophoridae), from the early Carnian/Julian of Polzberg bei Lunz, Austria (Triassic, 228-225 Ma). Our prior assumed 152 My as the minimum age and 225 My for the upper bound.

MCRA of Osteoglossomorpha (Fig. 1, node 7). The oldest crown osteoglossomorphs are several taxa from the Lycoptera assemblage of the Barremian of China (Early Cretaceous, 130 Ma), such as the Hiodontidae *Yanbiania wangqingica* [9]. To establish an upper boundary we used the elopomorph *Anaethalion*, from the late Kimmeridgian lithographic limestone (Jurassic, 152 Ma) [8]. Our prior assumed 130 My as the minimum age, and 152 My as the upper bound.

MCRA of Elopomorpha (Fig. 1, node 8). The oldest crown elopomorph is the albulid *Albuloideorum ventralis*, from the Early Hauterivian (Jurassic/Cretaceous border, 135 Ma) [10]. The oldest stem elopomorph is *Anaethalion*, from the late Kimmeridgian lithographic limestone of Nusblingen, Germany, and Cerin, France (Jurassic, 152 Ma) [8]. Our prior assumed 135 My as the minimum age, and 152 My for the upper bound.

MCRA of Ostarioclupeomorpha (Ostariophysi vs Clupeomorpha) (Fig. 1, node 9). The oldest known crown ostarioclupeomorph fossil is *Tischlingerichthys viohli*, upper

Tithonian of Muhlheim, Bavaria, Germany (Jurassic, 149 Ma) [11]. To establish a 95% upper boundary we used the elopomorph *Anaethalion*, from the late Kimmeridgian lithographic limestone of Nusblingen, Germany, and Cerin, France (Jurassic, 152 Ma) [8]. Our prior assumed 149 My as the minimum age, and 152 My for the upper bound.

Pellona* vs *Alosa (Fig. 1, node 10). The oldest fossil that can be assigned to this clade is *Gasteroclupea branisai*, from the Maastrichtian of the El Molino Formation, Cayara, Bolivia (Late Cretaceous, 72-67 Ma) [12]. A number of clupeiform fossils from the Lower Cretaceous (e.g., '*Clupea*' spp.; *Ellimichthys longicostatus*; *Paraclupea chetungensis*; 130-125 Ma) provide us with the upper boundary [13]. The prior assumed a minimum age of 67 My, and an upper boundary of 125 My.

MCRA of Ostariophysi (Fig. 1, node 11). The oldest crown ostariophysans are the Chanidae *Gordichthys* and *Rubiesichthys* from the Berriasian/Barremian (Jurassic/ Cretaceous, 145-125 Ma) [14]. The ostarioclupeomorph *Tischlingerichthys viohli*, from upper Tithonian of Muhlheim, Germany (Jurassic, 149 Ma), was used to establish an upper boundary. Our prior thus assumed 125 My as the minimum age, and 149 My as the 95% upper boundary.

MCRA of Characiformes (Fig. 1, node 12). *Santanichthys diasii* from the Santana formation in Brazil, Albian (Early Cretaceous, 112-100 Ma) is the oldest stem characiform [15]. The oldest crown characiforms are various Serrasalminae *indet*. and Tetraogonopterinae *indet*. from the middle Maastrichtian of the El Molino Formation,

Bolivia (Late Cretaceous, 69-68 Ma) [16]. Our prior assumed 68 My as the minimum age, and 100 My as the 95% upper boundary.

MCRA of Cyprinidae (Fig. 1, node 13). *Parabarbus* spp. from the Ypresian of the Obailinskaya Formation, Zaissan Basin, Kazakhstan (early-middle Eocene, 51-49 Ma) is the oldest crown cyprinid [17] and provided the minimum age for the cyprinid calibration. The stem characiform *Santanichthys diasii* from the Santana formation in Brazil, Albian (Early Cretaceous, 112-100 Ma) was used to set the 95% upper boundary [15]. Our prior assumed 49 My as the minimum age, and 100 My as the 95% upper boundary.

MCRA of Siluriformes (Fig. 1, node 14). The oldest fossils that have been assigned to the crown Siluriformes are incomplete remains of Ariidae from the Campanian-Maastrichtian of Argentina (Late Cretaceous, 73 Ma) [18]. The oldest siluriforms are *incertae sedis* materials from the Adamantina Formation of Brazil, Turonian-Santonian (Late Cretaceous, 93.6-83.5 Ma) [19]. Our prior thus assumed 73 My as the minimum age, and 83.5 My as the upper boundary.

MCRA of Bagridae (Fig. 1, node 15). The oldest bagrid fossils, *Eomacrones wilsoni*, *Nigerium gadense*, *Nigerium wurnoënse,* have all been assigned to deposits in Niger of Paleocene age (~59 Ma) [18]. Our prior assumed 59 My as the minimum age, and 73 My, age assigned to the crown siluriforms, as the upper boundary [18].

MCRA of Callichthyidae (Fig. 1, node 16). The oldest callichthyid, *Corydoras revelatus*, dates from the Thanetian of Argentina *(*Paleocene, ~55 Ma) [18]. Our prior assumed 55 My as the minimum age, and 73 My, age assigned to the crown siluriforms, as the upper boundary [18].

MCRA of Ictaluridae (Fig. 1, node 17). *Astephus* sp. from the Thanetian of Wyoming (Paleocene, 56 Ma) provides minimum age for this group [18]. Our prior assumed 55 My as the minimum age, and 73 My, age assigned to the crown siluriforms, as the upper boundary [18].

MCRA of Argentiniformes (Fig. 2, node 18). The argentiniform *Nybelinoides brevis* from the Barremian-Aptian of Bernissart, Belgium (Early Cretaceous, 127-124 Ma) marks the earliest appearance of the crown argentiniforms [20]. *Leptolepides sprattiformis* (Orthogonikleithridae) from the late Kimmeridgian lithographic limestone of Cerin, France (Jurassic, 152 Ma) is the oldest euteleost [11]. Our prior assumes 124 My as the minimum age, and 152 My as the upper boundary

MCRA of Osmeridae (Fig. 2, node 19). *Speirsaenigma lindoei*, from the Thanetian of the Paskapoo Formation, Alberta, Canada (Paleocene, ~58.7 Ma) is the oldest osmerid fossil [21]. The otoliths from the Late Santonian of Font de las Bagasses, Spain (Late Cretaceous, 87-84 Ma) are used to set the upper boundary [22]. Our prior assumes 58.7 My as the minimum age, and 84 My as the upper boundary.

MCRA of Galaxiidae (Fig. 2, node 20). *Stompooria rogersmithi* from the Maastrichthian of South Africa (late Cretaceous, 70 Ma) [23] indicates the appearance of the crown galaxiids. The Argentinidae *Nybelinoides brevis* from the Barremian-Aptian of Belgium (Early Cretaceous, 127-124 Ma) is used to establish the upper boundary [20]. The prior thus assumes 70 My as the minimum age, and 124 My for the 95% upper bound.

MCRA of Esociformes (Fig. 2, node 21). The oldest Esociformes is *Estesesox foxi* from the Campanian of the Milk River Fomation, Alberta, Canada (Late Cretaceous, 85 Ma) [24]. *Leptolepides sprattiformis* (Orthogonikleithridae) from the late Kimmeridgian lithographic limestone of Cerin, France (Jurassic, 152 Ma) is the oldest euteleost [11]. Our prior assumes 85 My as the minimum age, and 152 My as the upper boundary.

MCRA of Salmoniformes (Fig. 2, node 22). The origin of Salmoniformes is indicated by *Helgolandichthys schmidi*, from the early Aptian of Tock, Helgolang, Germany (Early Cretaceous, 125 Ma) [25]. Due to uncertainty on the placement of this taxon we assign its age to the upper boundary. The minimum age of the crown salmoniforms in our prior is that of *Eosalmo driftwoodensis* from the Lutetian (Eocene, 48 Ma) [26]. Our prior assumes 48 My as the minimum age, and 125 My as the upper boundary.

MCRA of Aulopiformes (Fig. 2, node 23). An undescribed alepisauroid from the early Barremian of Alcaine, Oliete subbasin, NE Spain (Early Cretaceous, 130-128 Ma) [27] is used to mark the appearance of the earliest aulopiforms. This taxon is a stem to the crown alepisauriforms present in our analysis, so we assign its age to the upper

boundary. The minimum age of the crown alepisauriforms is assigned on the basis of the fossils *Nematonotus* spp. (Aulopodidae) and *Acrognathus dodgei* (Chlorophthtalmidae) from the Cenomanian of Hakel, Lebanon (Late Cretaceous, 98-96 Ma) [13]. The prior assumed 96 My as the minimum age, and 128 My as the upper boundary.

MCRA of Acanthomorpha (Fig. 2, node 24). The fossil otoliths assigned to the “genus *Acanthomorphorum*" *forcallensis* from the early Aptian of the Maestrazgo, Castellon Province, Spain (Early Cretaceous, 124-122 Ma) [28]. Various Beryciformes (e.g., *Hoplopteryx*, *Trachichthyoides*) from the Cenomanian of the Lower Chalk, SE England (Late Cretaceous, 99 Ma) represent the oldest record of crown acanthomorphs [13]. The prior assumed a minimum age of 99 My, and an upper boundary of 122 My.

MCRA of Beryciformes (Fig. 2, node 25). Various Beryciformes (e.g., *Hoplopteryx*, *Trachichthyoides*) from the Cenomanian of the Lower Chalk, SE England (Late Cretaceous, 99 Ma) represent the appearance of the crown [13]. The fossil otoliths assigned to the “genus *Acanthomorphorum*" *forcallensis* from the early Aptian of the Maestrazgo, Castellon Province, Spain (Early Cretaceous, 124-122 Ma) [28] is used to identify the upper boundary. The prior assumed a minimum age of 99 My, and an upper boundary of 122 My.

MCRA of Lampridiformes (Fig. 2, node 26). *Nardovelifer altipinnis* is the oldest crown lampridiform currently known, from the Late Campanian of Nardó, Italy (Late

Cretaceous, 75-70 Ma) [29]. Undescribed taxa from the Cenomanian of Hakel, Lebanon (Late Cretaceous, 97-98 Ma) [30] represent the earliest appearance of lampridiforms in the fossil record. The prior assumed 70 My as the minimum age, and 98 My as the upper boundary.

MCRA of Ophiididae (Fig. 2, node 27) The otoliths of "genus *Ophidiidarum*" *cavatus* from the early Maastrichtian of Coon Creek Tongue, Blue Spring, Mississippi (Late Cretaceous, 70-68 Ma) marks the appearance of the group in the fossil record [31]. The Beryciformes (e.g., *Hoplopteryx*, *Trachichthyoides*) from the Cenomanian of the Lower Chalk, SE England (Late Cretaceous, 99 Ma) are used to establish an upper boundary [13]. The prior assumed a minimum age of 68 My, and an upper boundary of 99 My.

Fundulidae vs Poeciliidae (Fig. 3, node 28). This split is dated by Poeciliidae indet. from the Thanetian of the Maiz Gordo Formation, Argentina (Paleocene, 58-55 Ma) [32]. The Beryciformes (e.g., *Hoplopteryx*, *Trachichthyoides*) from the Cenomanian of the Lower Chalk, SE England (Late Cretaceous, 99 Ma) are used to establish an upper boundary [13]. The prior assumed a minimum age of 55 My, and an upper boundary of 99 My.

MCRA of Channoidea (Fig. 2, node 29). *Eochanna chorlakiensis* from the Ypresian and Lutetian (Middle Eocene, 49-48 Ma) marks the appearance of the clade [33]. The otoliths assigned to the "genus *Epigonidarum*" *weinbergi* from the Coniacian of Tiefe Gosau, Ennstaler Alpen, Austria (Late Cretaceous, 89-84 Ma) are used to establish an

upper boundary [34]. The prior assumed 48 My as the minimum age, and 84 My as the upper boundary.

MCRA of Cichlidae (Fig. 3, node 30). *Mahengechromis* spp. From the early Lutetian of the Mahenge paleolake, Tanzania, Africa (Eocene, 46 Ma) [35] is the oldest taxon that can be assigned to the crown cichlids. The otoliths assigned to the "genus *Epigonidarum*" *weinbergi* from the Coniacian of Tiefe Gosau, Ennstaler Alpen, Austria (Late Cretaceous, 89-84 Ma) are used to establish an upper boundary [34]. The prior assumed 46 My as the minimum age, and 84 My as the upper boundary.

MCRA of African Cichlidae (Fig. 3, node 31). *Heterochromis* fossils from the late Chattian Ad Daib, Baid Formation, Tihamat Asir, SW Saudi Arabia (Oligocene, 23.3 Ma) are used to date this clade [36]. The otoliths assigned to the "genus *Epigonidarum*" *weinbergi* from the Coniacian of Tiefe Gosau, Ennstaler Alpen, Austria (Late Cretaceous, 89-84 Ma) are used to establish an upper boundary [34]. The prior assumed 23.3 My as the minimum age, and 84 My as the upper boundary.

MCRA of Gerreidae (Fig. 3, node 32). *Gerres latidens* from the Ypresian of the Wittering Formation, London Clay, Southern England, UK (Eocene, 54-52 Ma) marks the appearance of the clade [37]. The otoliths assigned to the "genus *Epigonidarum*" *weinbergi* from the Coniacian of Tiefe Gosau, Ennstaler Alpen, Austria (Late Cretaceous, 89-84 Ma) are used to establish an upper boundary [34]. The prior assumed 52 My as the minimum age, and 84 My as the upper boundary.

MCRA of Gobiidae (Fig. 2, node 33). Gobiidae indet. From the Lutetian (Eocene, 48-40 Ma) is used to determine the minimum age [38]. The otoliths assigned to the "genus *Epigonidarum*" *weinbergi* from the Coniacian of Tiefe Gosau, Ennstaler Alpen, Austria (Late Cretaceous, 89-84 Ma) are used to establish an upper boundary [34]. The prior assumed 40 as the minimum age, and 84 as the upper boundary.

MCRA of Labridae (Fig. 3, node 34). Several taxa (e.g., *Eocoris bloti*, *Phyllopharyngodon longipinnis*) from the late Ypresian of Monte Bolca, Italy (Eocene, 50 Ma) are used to determine the minimum age of the crown labrids [39]. The otoliths assigned to the "genus *Epigonidarum*" *weinbergi* from the Coniacian of Tiefe Gosau, Ennstaler Alpen, Austria (Late Cretaceous, 89-84 Ma) are used to establish an upper boundary [34]. The prior assumed 50 My as the minimum age, and 84 My as the upper boundary.

MCRA of Moronidae (Fig. 3, node 35). *Morone* sp. from the Late Campanian of Coffee Sand, Northeastern Mississippi (Late Cretaceous, 74-73 Ma) is the oldest fossil assigned to moronids [40]. Due to the uncertainty of the phylogenetic relationships within the “Perciformes”, it is not currently known which is the sister group of the Moronidae. The otoliths assigned to the "genus *Epigonidarum*" *weinbergi* from the Coniacian of Tiefe Gosau, Ennstaler Alpen, Austria (Late Cretaceous, 89-84 Ma), however, indicate the earliest appearance of taxa belonging to the clade of “Perciformes” that likely includes the Moronidae, and are thus used to establish an

upper boundary [34]. The prior assumed 74 My as the minimum age, and 84 My as the upper boundary.

MCRA of Pomacentridae (Fig. 2, node 36). Several taxa (e.g., *Palaeopomacentrus orphae*, *Lorenzichthys olihan*) from the late Ypresian of Monte Bolca, Italy (Eocene, 50 Ma) are used to determine the minimum age of the crown pomacentrids [41]. The otoliths assigned to the "genus *Epigonidarum*" *weinbergi* from the Coniacian of Tiefe Gosau, Ennstaler Alpen, Austria (Late Cretaceous, 89-84 Ma) are used to establish an upper boundary [34]. The prior assumed 50 My as the minimum age, and 84 My as the upper boundary

MCRA of Pleuronectiformes (Fig. 2, node 37). The otolith of several taxa assigned to the families Bothidae, Citharidae, and Psettodidae from the Late Ypresian of Tuilerie de Gan, Argile de Gan, Gan, Pyrénées-Atlantique, France (Eocene, 52-51 Ma) indicate the appearance of the crown [42]. The Beryciformes (e.g., *Hoplopteryx, Trachichthyoides*) from the Cenomanian of the Lower Chalk, SE England (Late Cretaceous, 99 Ma) are used to establish an upper boundary [13]. The prior assumed a minimum age of 51 My, and an upper boundary of 99 My.

MCRA of Tetraodontiformes (Fig. 3, node 38). This oldest fossil that can be assigned to the crown tetraodontiforms is the stem balistoid *Moclaybalistes danekrus*, from the Moclay deposits of Denmark (Palaeocene, 59 Ma) [43]. The oldest tetraodontiform fossil is *Plectocretacicus clarae*, from the Cenomanian of Lebanon (Cretaceos, 98 Ma) [44].

We thus assigned a lower bound of 59 My and an upper bound of 98 My to this calibration.

***MCRA* of Balistoidea (Balistidae vs Monacanthidae)** (Fig. 3, node 39). The stem balistids *Balistomorphus*, which includes the species *B. orbiculatus*, *B. ovalis* and *B. spinosus*, and *Oligobalistes robustus* are all from the early Oligocene of, respectively, Switzerland and Caucasus (35 Ma) [43]. They provide a minimum age estimate for the split between balistids and monacanthids. Our prior assigns a minimum age of 35 My to this calibration, and an upper bound of 50 My to this calibration (reflecting the appearance of several other tetraodontiform families in Monte Bolca) [43].

MCRA of Ostracioidea (Aracanidae vs. Ostraciidae) (Fig. 3, node 40). *Eolactoria sorbinii* is a stem ostraciid, and *Proaracana dubia* is a stem aracanid [43]. Both are from the Ypresian of Monte Bolca, Italy (middle Eocene, 50 Ma). We assigned a lower bound of 50 My and an upper bound of 70 My, likely age of crown tetraodontiforms, as derived from Alfaro et al. [45] to this calibration.

MCRA of Tetraodontoidea (Tetraodontidae vs. Diodontidae) (Fig. 3, node 41). Several stem diodontids, *Prodiodon erinaceus, Prodiodon tenuispiis*, *Heptadion echinus* and *Zignodon fornasieroae* [43], and the stem tetraodontid *Eotetraodon pygmaeus* [43, 46] are known from the Ypresian of Monte Bolca, Italy (middle Eocene, 50 Ma). We used this date as a lower bound and assigned an upper bound of 70 My, likely age of crown tetraodontiforms, as derived from [45] to the calibration.

MRCA of Tetraodontidae (Fig. 3, node 42). The fossil *Archaeotetraodon winterbottomi* [47] from the Oligocene of Caucasus, Russia, has been assigned to the crown Tetraodontidae, and provides a minimum age estimate of 35 Ma for the MRCA of the family [48]. We assigned an upper bound of 50 My (age of stem tetraodontids, see above) to this calibration.

MCRA of Caproidae (Fig. 3, node 43). *Eoantigonia veronensis* from the late Ypresian of Monte Bolca, Italy (Eocene, 50 Ma) marks the split between *Antigonia* and *Capros* [49]. The Beryciformes (e.g., *Hoplopteryx*, *Trachichthyoides*) from the Cenomanian of the Lower Chalk, SE England (Late Cretaceous, 99 Ma) are used to establish an upper boundary [13]. The prior assumed a minimum age of 50 My, and an upper boundary of 99 My.

MCRA of Zeiformes (Fig. 2, node 44). *Cretazeus rinaldii* from the Late Campanian of Nardó, Italy (Late Cretaceous, 72 Ma) marks the appearance of the crown zeiforms [50, 51]. The Beryciformes (e.g., *Hoplopteryx*, *Trachichthyoides*) from the Cenomanian of the Lower Chalk, SE England (Late Cretaceous, 99 Ma) are used to establish an upper boundary [13]. The prior assumed a minimum age of 72 My, and an upper boundary of 99 My.

References

1. Samson IJ, Smith MM, Smith MP: **Scales of thelodonts and shark-like fishes from the Ordovician of Colorado.** *Nature* 1996, **379**:628-630.
2. Zidek J: **Acanthodii.** In *The fossil record 2* Edited by Benton MJ. London: Chapman & Hall; 1993:587-590.
3. Conway Morris S: **The Crucible of Creation: The Burgess Shale and the Rise of *Animals.*** NY, USA: Oxford University Press; 2000.
4. Cappetta H, Duffin C, Zidek J: **Chondrichthyes.** In *The fossil record 2* Edited by Benton MJ. London: Chapman & Hall; 1993:593-609.
5. Marss T: ***Andreolepis* (Actinopterygii) in the upper Silurian of northern Eurasia.** *Proc Eston Acad Sc Geol* 2001, **50**:174-189.
6. Johanson Z, Long JA, Talent JA, Janvier J, Warren JW: **Oldest coelacanth, from the Early Devonian of Australia.** *Biol Letters* 2006, **2**:443–446.
7. Hurley IA, Mueller RL, Dunn KA, Schmidt KJ, Friedman M, Ho RK, Prince VE, Yang Z, Thomas MG, Coates MI: **A new time-scale for ray-finned fish evolution.** *Proc R Soc B* 2007, **274**: 489-498.
8. Arratia G: **Mesozoic halecostomes and the early radiation of teleosts.** In *Mesozoic fishes 3* Edited by Arratia GF, Tintori A. München: Verlag Dr. Friedrich Pfeil; 2004:279-315.
9. Li GQ, Wilson MVH, Grande L: **Review of *Eohiodon* (Teleostei: Osteoglossomorpha) from western North America, with a phylogenetic reassessment of Hiodontidae.** *J Paleo* 1997, **71**:1109-1124.

10. Weiler W: ***Palealbula ventralis* n. sp. (Pisces, Clupeiformes) aus dem Neocom (Unter Hauterive) von Engelbostei bei Hannover.** *Senckenbergiana Lethaea* 1971, **52**:1-3.
11. Arratia G: **Basal teleosts and teleostean phylogeny.** *Palaeo Ichthyologica* 1997, **7**:5-168.
12. Signeux J: ***Gasteroclupea branisai*, clupéidé nouveau du Crétacé supérieur de Bolivie.** *Bull Mus Nat Hist Nat Paris Sér 4* 1964, **39**:290-297.
13. Patterson C: **Osteichthyes: Teleostei.** In *The fossil record 2* Edited by Benton, M.J. London: Chapman & Hall; 1993:621-656.
14. Poyato-Ariza FJ: **The phylogenetic relationships of *Rubiesichthys gregalis* and *Gordichthys conquensis* (Ostariophysi, Chanidae), from the Early Cretaceous of Spain.** In *Mesozoic Fishes - Systematics and Paleoecology,* Edited by Arratia G, Viohl G. München: Verlag Dr. Friedrich Pfeil; 1996:329-348.
15. Filleul A, Maisey JC: **Redescription of *Santanichthys diasii* (Otophysi, Characiformes) from the Albian of the Santana Formation and Comments on its implications for Otophysan relationships.** *Am Mus Novit* 2004, **3455**:1-21.
16. Gayet M, Marshall LG, Sampere T, Meunier F, Cappetta H, Rage JC: **Middle Maastrichtian vertebrates (fishes, amphibians, dinosaurs and other reptiles, mammals) from Bajcha Bata (Bolivia). Biostratigraphic, paleoecologic and paleobiogeographic implications.** *Palaeogeog, Palaeoclim, Palaeoecol* 2001, **169**:39-68.
17. Sytchevskaya EY: **Paleogene freshwater fish fauna of the USSR and Mongolia.** *Trans Joint Sov-Mong Paleo Exped* 1986, **19**:1-157.

18. Gayet M, Meunier F: **Palaeontology and palaeobiogeography of catfishes.** In *Catfishes* Edited by Arratia G, Kapoor BG, Chardon M, Diogo R. Enfield, NH: Science Publisher; 2003:491-522.

19. Bertini RJ, Marshall LG, Gayet M, Brito P: **Vertebrate faunas from the Adamantina and Marilia formations (Upper Bauru Group, Late Cretaceous, Brazil) in their stratigraphic and paleobiogeographic context.** *Neu Jahrb Geol Palao, Abhand* 1993, **188**:71-101.

20. Taverne L: **Sur *Pattersonella formosa* (Traquair R.H. 1911) et *Nybelinoides brevis* (Traquair R.H. 1911), Téléostéens Salmoniformes Argentinoides du Wealdien inférieur de Bernissart, Belgique, précédemment attribués au genre Leptolepis Agassiz L. 1832.** *Bull Inst Roy Sci Nat Belg* 1982, **54**:1-27.

21. Wilson MVH, Williams RRG: **New Paleocene genus and species of smelt (Teleostei: Osmeridae) from freshwater deposits of the Paskapoo Formation, Alberta, Canada, and comments on osmerid phylogeny.** *J Vert Paleo* 1991, **11**:434-451.

22. Nolf D: **Fish otoliths from the Santonian of the Pyrenean faunal province, and an overiview of all otolith-documented North Atlantic Late Cretaceous.** *Bull Inst Roy Sci Nat Belg, Sci Terre* 2003, **73**:155-151.

23. Anderson ME: **A Late Cretaceous (Maastrichtian) galaxiid fish from South Africa.** *JLB Smith Inst Ichthyol Spec Publ* 1998, **60**

24. Wilson MVH, Brinkman DB, Neuman AG: **Cretaceous Esocoidei (Teleostei): Early radiation of the pikes in North American fresh-waters.** *J Paleo* 1992, **66**: 839-846.

25. Taverne L: **Les actinopterygiens de l'Aptien Inférieur (Tock) d'Helgoland.** *Mit Geol-Palao Ins Univ Hamb* 1981, **51**:43-82.

26. Wilson MVH, Li GQ: **Osteology and systematic position of the Eocene salmonid *Eosalmo driftwoodensis* Wilson from western North America.** *Zool J Linn Soc* 1999, **125**:279-311.

27. Kriwet J: **Lancetfish teeth (Neoteleostei; Alepisauroidei) from the Early Cretaceous of Alcaine, NE Spain.** *Lethaia* 2003, **36**:323-332.

28. Nolf D: **Otolithes de poissons aptiens du Maestrazgo (province de Castellon, Espagne orientale).** *Bull Inst Roy Sci Nat Belg, Sci Terre* 2004, **74**:101-120.

29. Sorbini C, Sorbini L: **The Cretaceous Fishes of Nardo. 10◦. *Nardovelifer altipinnis*, gen. et sp. nov. (Teleostei, Lampridiformes, Veliferidae).** *St Ric Giac Terz Bolca* 1999, **8**:11–27.

30. Gayet M, Belouze A, Abi Saad P: **Les Poissons Fossiles.** Editions Désiris, Méolans-Ravelm; 2003.

31. Nolf D, Stringer GL: **Cretaceous fish otoliths - a synthesis of the North American record.** In *Mesozoic fishes - Systematics and paleoecology* Edited by Arratia G, Viohl G. Munchen: Verlag Dr. Friedrich Pfeil; 1996:433-456.

32. Cione AL: **Los peces continentales del Cenozoico de Argentina.** *Act Cong Argent Paleo Bioest* 1986, **4**:101-106.

33. Roe LJ: **Phylogenetic and ecological significance of Channidae (Osteichthyes, Teleostei) from the Early Eocene Kuldana Formation of Kohat, Pakistan.** *Contrib Mus Paleo, Univ Michigan* 1991, **28**:93-100.

34. Sieber R, Weinfurter E: **Otolithen aus Tiefen Gosauschichten, Osterreichs.** *Ann Naturhhist Mus Wien* 1967, **71**:353-361.

35. Murray AM: **The oldest fossil cichlids (Teleostei: Perciformes): indication of a 45 million-year-old species flock.** *Proc Biol Sci* 2001, **268**:679-684.

36. Micklich N, Roscher B: **Neue Fishfunde aus der Baid Formation (Oligozan; Tihamat Asir, SW Saudi Arabia).** *Neu Jahrb Geol Palao, Abhand* 1990, **180**:139-175.

37. Stinton FC: **Fish otoliths from the English Eocene, IV.** *Palaeo Soc, Monog* 1980, **4**:191-258.

38. Gaudant J: **Mise en évidence des plus anciens Gobioidei (Poissons téléostéens) connus dans le Lutétien inférieur marin de Catalogne (Espagne).** *Comptes Rendus Acad Sci, Paris II* 1996, **322**:71-76.

39. Bannikov AF, Sorbini L: ***Eocoris bloti*, a new genus and species of labrid fish (Perciformes, Labroidei) from the Eocene of Monte Bolca.** *St Ric Giac Terz Bolca* 1990, **6**:133-148.

40. Nolf D, Dockery DT: **Fish otoliths from the Coffee Sand (Campanian) of Northeastern Mississippi.** *Missis Geol* 1990, **10**:1-14.

41. Bellwood DR: **Fossil pharyngognath fishes from Monte Bolca, Italy, with a description of a new pomacentrid genus and species.** *St Ric Giac Terz Bolca* 1999, **8**:207-217.

42. Nolf D: **Les otolithes de téléostéens éocènes d'Aquitaine et leur intéret stratigraphique.** *Mém Acad Roy Belg, Cl Sci* 1988, **19**:1-147.

43. Tyler JC, Santini F: **Review and reconstructions of the tetraodontiform fishes from the Eocene of Monte Bolca, Italy, with comments on related Tertiary taxa.** *St Ric Giac Ter Bolca, Mus Civ St Nat Verona* 2002, **9**:47-119.

44. Tyler JC, Sorbini L: **New superfamily and three new families of tetraodontiform fishes from the Upper Cretaceous: the earliest and most morphologically primitive plectognaths.** *Smith Contrib Paleob* 1996, **82**:1-59.

45. Alfaro ME, Santini F, Brock CD: **Do reefs drive diversification in marine teleosts? Evidence from the pufferfishes and their allies (Order Tetraodontiformes).** *Evolution* 2007, **61**:2104-2126.

46. Tyler JC, Mirzaie M, Nazemi A: **A new genus and species of basal tetraodontoid puffer fish from the Oligocene of Iran, related to the Zignoichthyidae (Tetraodontiformes).** *Boll Mus Civ Stor Nat Verona* 2006, **30**:49-58.

47. Tyler JC, Bannikov AF: **A new genus of fossil pufferfish (Tetraodontidae: Tetraodontiformes) based on a new species from the Oligocene of Russia and a referred species from the Miocene of Ukraine.** *Proc Biol Soc Washington* 1994, **107**:97-108.

48. Santini F, Tyler JC: **A phylogeny of the families of fossil and extant tetraodontiform fishes (Acanthomorpha, Tetraodontiformes), Upper Cretaceous to Recent.** *Zool J Linn Soc* 2003, **139**:565-617.

49. Baciu D-S, Bannikov AF, Tyler JC: **Revision of the fossil fishes of the family Caproidae (Acanthomorpha).** *Misc Paleont, St Ric Giac Terz Bolca* 2005, **11**

50. Tyler JC, Bronzi P, Ghiandoni A: **The Cretaceous fishes of Nardo 11°. A new genus and species of Zeiformes, *Cretazeus rinaldii*, the earliest record for the order.** *Boll Mus Civ Stor Nat Verona* 2000, **24**:11-28.

51. Tyler JC, Santini F: **A phylogeny of the fossil and extant zeiform-like fishes, Upper Cretaceous to Recent, with comments on the putative zeomorph clade (Acanthomorpha).** *Zool Scripta* 2005, **34**:157–175.
